# Sequestration of Voriconazole and Vancomycin Into Contemporary Extracorporeal Membrane Oxygenation Circuits: An *in vitro* Study

**DOI:** 10.3389/fped.2020.00468

**Published:** 2020-08-27

**Authors:** Genny Raffaeli, Giacomo Cavallaro, Karel Allegaert, Birgit C. P. Koch, Fabio Mosca, Dick Tibboel, Enno D. Wildschut

**Affiliations:** ^1^Intensive Care and Department of Pediatric Surgery Erasmus MC–Sophia Children's Hospital, University Medical Center Rotterdam, Rotterdam, Netherlands; ^2^Department of Clinical Sciences and Community Health, University of Milan, Milan, Italy; ^3^Fondazione IRCCS Ca' Granda Ospedale Maggiore Policlinico, NICU, Milan, Italy; ^4^Department of Development and Regeneration and Department of Pharmaceutical and Pharmacological Sciences, KU Leuven, Leuven, Belgium; ^5^Hospital Pharmacy, Erasmus MC, Rotterdam, Netherlands

**Keywords:** extracorporeal membrane oxygenation, pharmacokinetics, pharmacology, antibiotics, infection, antifungals, drug disposition

## Abstract

**Background:** Bacterial and fungal infections are common and often contribute to death in patients undergoing extracorporeal membrane oxygenation (ECMO). Drug disposition is altered during ECMO, and adsorption in the circuit is an established causative factor. Vancomycin and voriconazole are widely used, despite the lack of evidence-based prescription guidelines.

**Objective:** The objective of this study was to determine the extraction of voriconazole and vancomycin by the Xenios/Novalung ECMO circuits.

**Methods:** We have set up nine closed-loop ECMO circuits, consisting of four different *iLAActivve*^®^ kits for neonatal, pediatric, and adult support: three *iLA-ActivveMiniLung*^®^ *petite* kits, two *iLA-ActivveMiniLung*^®^ kits, two *iLA-ActivveiLA*^®^ kits, and two *iLA-Activve X-lung*^®^ kits. The circuits were primed with whole blood and maintained at physiologic conditions for 24 h. Voriconazole and vancomycin were injected as a single-bolus age-related dose into the circuits. Pre-membrane (P2) blood samples were obtained at baseline and after drug injection at 2, 10, 30, 180, 360 min, and 24 h. A control sample at 2 min was collected for spontaneous drug degradation testing at 24 h.

**Results:** Seventy-two samples were analyzed in triplicate. The mean percentage of drug recovery at 24 h was 20% for voriconazole and 62% for vancomycin.

**Conclusions:** The extraction of voriconazole and vancomycin by contemporary ECMO circuits is clinically relevant across all age-related circuit sizes and may result in reduced drug exposure *in vivo*.

## Introduction

Critically ill patients on extracorporeal membrane oxygenation (ECMO) are at high risk of serious infections, with rates of 15.4 per 1,000 ECMO days across all age groups (1). As the mortality of ECMO patients facing an infective event increases up to 68%, a timely and adequate antimicrobial therapy is essential to improve outcomes (1–3).

Vancomycin is widely used as the first-line treatment for both the empiric and targeted treatment of blood stream infections caused by methicillin-resistant *Staphylococcus aureus* (MRSA), coagulase-negative staphylococci (CONS), and ampicillin-resistant enterococci (1, 4–6). Although bacteria remain the most common causative agents, fungi contribute to the healthcare burden being the second cause of nosocomial infection during ECMO (1), with a prevalence ranging from 0.04 to 5%, and differences based upon age and timing of infection (2, 5). Voriconazole is a second-generation triazole and a synthetic derivative of fluconazole, acting against *Candida* and *Aspergillus* spp., which are the most frequently isolated fungi in ICU (7). Indeed, the azole class is pivotal in the prevention and treatment of invasive fungal infections in critically ill patients (7, 8). While the pharmacology of fluconazole, which is the recommended first-line treatment against *Candida* spp., has been extensively addressed in the pediatric ECMO population (9–11), scanty evidence is available for voriconazole, a first-line treatment for invasive aspergillosis (7, 12, 13).

Besides the choice of the proper agent, the selection of the right dose to attain target exposure is crucial for therapeutic success (14). Drug dosing is known to be complex in critically ill patients, and it is further complicated in specific populations, in which pathology-related factors sum up to physiologic age-specific PK changes (15). The need for an extracorporeal support adds interfering mechanisms, which are responsible for further PK variability (16).

ECMO is increasingly spreading, especially across adult intensive care settings, to support potentially reversible cardiac or respiratory failure, unresponsive to conventional treatment (17). This procedure requires the addition of fluids and exogenous blood products to prime the circuit, thereby increasing the circulating volume. As a consequence of hemodilution, priming compositions, and organ dysfunction, both the volume of distribution (Vd) and clearance (Cl) of drugs are altered (18). A certain degree of sequestration into the circuit may be expected based on the physicochemical characteristics of a drug, such as molecular size, plasma protein binding, degree of ionization at physiological pH, and lipophilicity, as all are based on the characteristics of the circuits and oxygenators (18–21). Moreover, the exposure of blood to the exogenous surface triggers a systemic inflammatory response, further contributing to altered antimicrobial disposition and overall therapeutic efficacy (16, 20, 22).

Despite the wide use of antimicrobial agents during ECMO (23), PK data related to newer systems are limited and heterogeneous, resulting in a poor level of evidence-based pharmacotherapy (22, 24). Previous pharmacological knowledge (25–27) needs to be updated according to the recent advances of extracorporeal technology to prevent altered antimicrobial exposure during ECMO. Furthermore, the optimization of pharmacotherapy is required to limit the burden of microbial resistance (28, 29).

The aim of this study was to provide new insights into the disposition of voriconazole and vancomycin in contemporary neonatal, pediatric, and adult ECMO circuits.

## Materials and Methods

The study was carried out at the Intensive Care Unit of the Sophia Children's Hospital—ECMO Center, Erasmus MC in Rotterdam, the Netherlands. The need for the institutional review board approval was waived as ethical approval was not required, according to the Dutch Law of research on human subjects. In particular, the fact that no patients were used in this *in vitro* study determined this decision. Study circuits were provided by the Xenios® company as part of an unrestricted research grant.

### *In vitro* ECMO Model

The *in vitro* ECMO model has been previously published (28). Here, we present a brief overview of the setting. We have set up nine different *in vitro* ECMO circuits, consisting of four *iLAActivve*^®^ kits: *iLA-ActivveMiniLung*^®^ petite kits (*n* = 3), *iLA-ActivveMiniLung*^®^ kits (*n* = 2), *iLA-ActivveiLA*^®^ kits (*n* = 2), and *iLA-Activve X-lung*^®^ kits (*n* = 2). They were made up of hollow fiber oxygenators with a diffusion membrane for neonatal, pediatric, and adult patients. All circuits were whole blood primed, connected to a 100-ml reservoir bag and run for 24 h at a flow rate of 500 ml/min (neonatal), 700 ml/min (infant), 2.5 L/min (pediatric), and 3.5 L/min (adult) with an estimated circuit volume of 225, 280, 360, and 400 ml, respectively. Anticoagulation was provided by administering a bolus of heparin 500 UI to maintain activated clotting times over 200 s. Experimental conditions (temperature, pH, hematocrit) have been maintained stable throughout the study period, as previously described (30).

### Drug Selection and Administration

We selected voriconazole and vancomycin for their clinical relevance in the Intensive Care Unit (ICU) practice and as illustrative examples of different chemical properties and their drug classes. Dosing for neonatal circuits was based on a standardized neonatal weight (3.5 kg), while for the other systems, we have increased the dose in proportion to the rise of volume to achieve similar theoretical concentrations. We injected voriconazole 35 mg/40 mg/52.5 mg/68 mg (neonatal/infant/pediatric/adult) followed by vancomycin 45 mg/54 mg/67.5 mg/91.8 mg through a line connected to the tubing right after the reservoir bag (P1), with 5-s intervals in-between injections. We flushed the line with 1 ml of physiological saline solution (0.9%) after voriconazole administration to avoid crystallization or pooling effects.

### Samples

A line attached to the tubing prior to the reservoir bag was used as a pre-membrane (P2) sample-drawing site. For each system, we collected samples before a single-bolus drug administration (T0) and during the circuit run at the following time intervals: 2 (T2), 10 (T10), 30 (T30), 180 (T180), 360 (T360) min, and 24 h (T24) after injection. At T2, a control sample was collected and analyzed at 24 h (T2-24) to determine spontaneous drug degradation. The T2–24 samples were stored in ethylenediaminetetraacetate (EDTA) sample tubes, which were maintained at the same temperature as the tested systems until 24 h. For all other samples (T0–T24), whole blood was collected in polypropylene EDTA tubes and stored at 4°C for a maximum of 12 h until further processing. Afterward, all blood samples were centrifuged (3,000 rounds/min for 6 min), and the plasma supernatant was transferred to labeled polypropylene cryogenic vials with polyethylene screw caps (Nalgene Labware, Rochester, NY, USA). Plasma samples were maintained at −80°C until measurement.

### Quantification of Drugs in Plasma Samples

Voriconazole and vancomycin were analyzed by means of ultrafast liquid chromatography–mass spectrometry (LC-MS/MS) in the pharmacy laboratory of Erasmus MC, as previously described (30) with regard to voriconazole or a validated immune-assay (Architect, Abbott) for vancomycin. Methods were validated according to the US Food and Drug Administration guidelines for bioanalytical method validation (31).

Reference values are reported [including lower limit of quantification (LLOQ) and upper limit of quantification (ULOQ)]. Intra- and inter-assay means were within 15% of the target range value, as requested by the FDA. For voriconazole, the linear calibration range was 0.1–10 ng/ml. For vancomycin, the linear calibration range was 0.6–50 mg/L.

### Spontaneous Drug Degradation and Expected Drug Levels

Spontaneous drug degradation was defined as the amount of drug left after 24 h of spontaneous decay, and it was calculated as a difference in drug recovery between T2 and T2–24. Expected blood drug levels were calculated, accounting for the amount of drug administered and the total circulating volume. Mean recovery of each circuit category at 24 h was corrected by the average drug spontaneous degradation.

### Data Analysis

**Figure 1:**
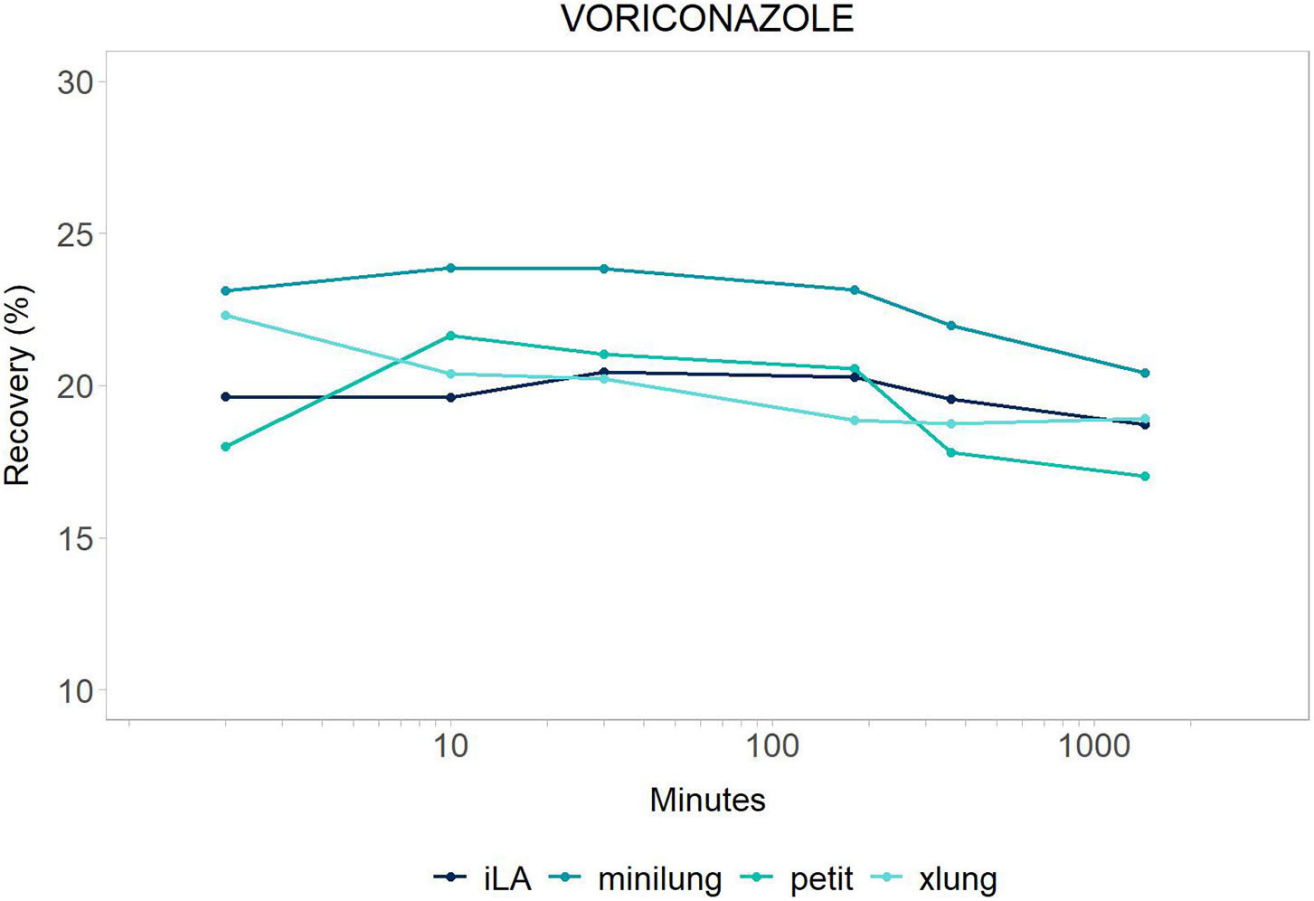
Voriconazole: mean drug recovery (%) plotted vs. time (min) over 24 h for antimicrobial across the four different categories of systems tested: *Minilung petite* (green), *MiniLung* (blue), *iLaActivve* (dark blue), and *XLung* (light blue).

**Figure 2:**
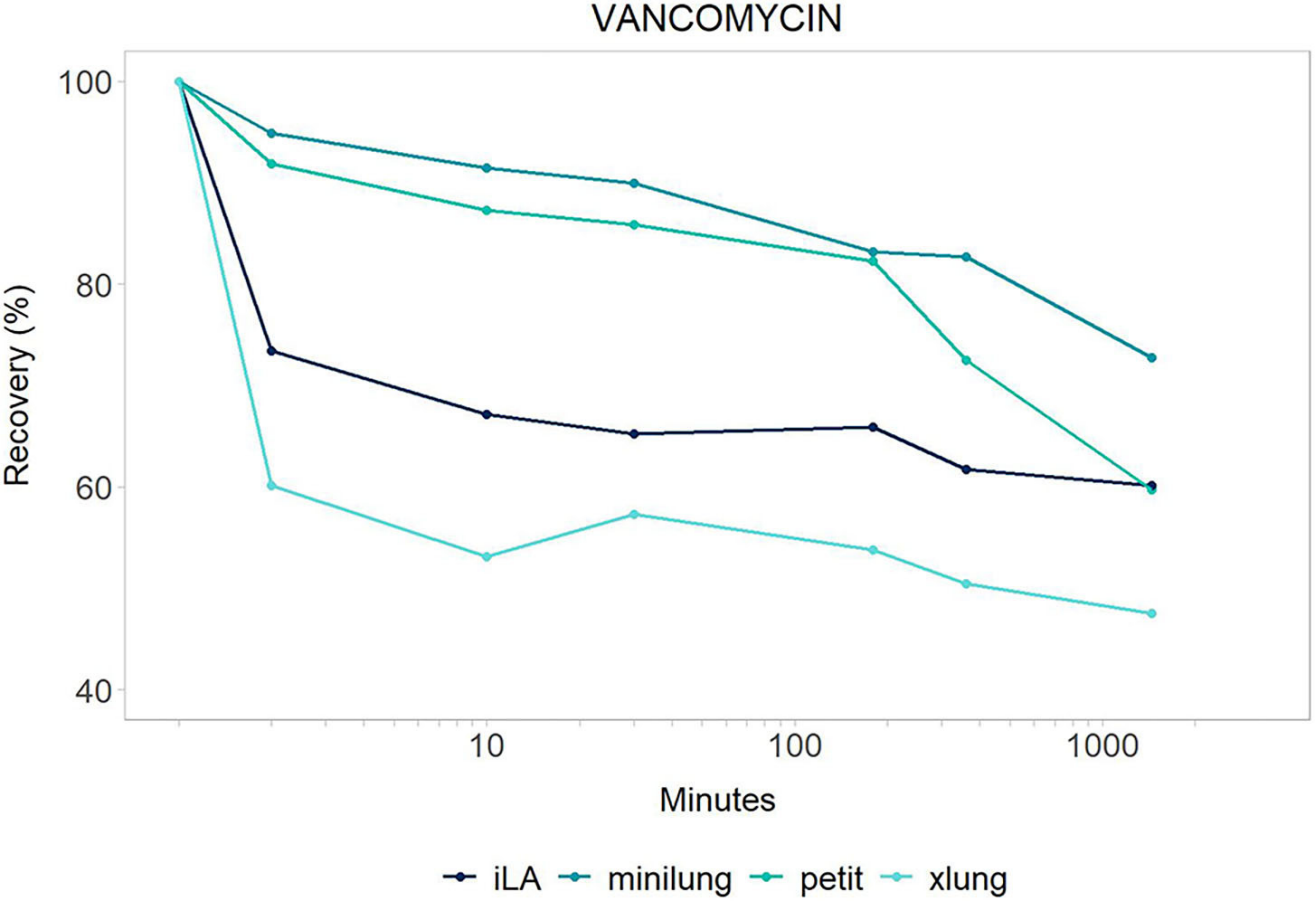
Vancomycin: mean drug recovery (%) plotted vs. time (min) over 24 h for antimicrobial across the four different categories of systems tested: *Minilung petite* (green), *MiniLung* (blue), *iLaActivve* (dark blue), and *XLung* (light blue).

We performed a comparative evaluation of drug disposition between four different ECMO circuits (MiniLung® petite: MLP, MiniLung®: ML, iLAActivve®: iLA, Xlung®). We calculated the average recovery corrected by spontaneous drug degradation at 2, 10, 30 min and 3, 6, and 24 h across all circuit categories. The trend over time is presented for the entire study period for each drug (see Figures 1, 2). Data are expressed as mean (±SD) or percentage. All analyses were performed using R version 3.4.3 (R Foundation for Statistical Computing, Vienna, Austria).

## Results

*In vitro* ECMO models were maintained under physiological conditions for 24 h. Throughout the experimental time, we did not experience any circuit complications. A total of 72 samples (eight for each circuit) were analyzed, in triplicate, for each drug. All baseline plasma samples were free of study drugs.

The recovery of drugs after a 24-h exposure to the extracorporeal circuit has been corrected for average drug spontaneous degradation, and it is graphically reported in Figures 1, 2. The mean percentage of drug recovery at 24 h was 20% for voriconazole and 62% for vancomycin. While the loss of voriconazole was similar across all age circuits, vancomycin levels decreased by 38% in the miniLung petite, by 25% in the minilung, by 38% in the ILA active, and by 50% in the XLung circuits (Table 1). Drug physicochemical properties, namely, lipophilicity and protein binding, were derived from the Drug Bank online database (32) and are summarized in Table 1.

**Table 1:** Mean drug recovery (mg/L; ±SD) after 24 h of circulation in the study testing circuits (r), corrected for spontaneous drug degradation compared to expected drug levels (e).

**Drug**	**e-MLP**	**r-MLP (*n* = 3)**	**e-ML**	**r-ML (*n* = 2)**	**e-iLA**	**r-iLA (*n* = 2)**	**e-XL**	**r-XL (*n* = 2)**	**Spontaneous degradation**	**LogP**	**PB**
VOR	140	24.9 (±3.5)	117.1	25 (±0.3)	134.7	26.3 (±0)	143.2	28.2 (±4.6)	0.8%	1.8	58
VAN	180	112 (±31.6)	177.1	133.2 (±49.7)	173.2	108.5 (±4,4)	193.7	96.5 (±13.5)	2.4%	−3.1	50

*iLA, iLAActivve® MLP, MiniLung® petite; ML, MiniLung® PB, protein binding; VAN, vancomycin; VOR, voriconazole; XL, Xlung®. LogP values and protein binding (PB) of study drugs are derived from Drug Bank*.

## Discussion

Suboptimal antimicrobial exposure may be associated with therapeutic failure, toxicity, antimicrobial resistance, and, ultimately, worsened patient outcomes (2, 3). In this *in vitro* study, we provide the equipment-related disposition of voriconazole and vancomycin in contemporary ECMO circuitry with *iLAActivve*, contributing insights into circuit- and drug-specific determinants of PK changes during ECMO. Voriconazole was largely sequestered in the circuits, with no distinction across the different circuit sizes. Vancomycin was absorbed slightly more in the neonatal (miniLung petite), pediatric (ILA active), and adult (XLung) circuits than in the infant ones (miniLung). Spontaneous drug decay over 24 h was limited for both study drugs.

We consider these drugs as representative for the pharmacological classes they belong to, thus extending the relevance of our findings.

As expected, based on its lipophilicity and high protein binding (32), voriconazole was largely sequestered in the systems, uniformly across all size groups. Our data confirm those by Mehta et al., who reported a dramatic loss of 60% of voriconazole within 3 h from administration, in an *ex vivo* silicone membrane-based model of blood primed circuit (33). Similarly, the altered PK profile of voriconazole has been observed in pediatric and adult case series (34, 35), showing a drop of voriconazole plasma levels immediately after ECMO initiation or membrane change (36). Higher initial loading and daily doses have been suggested, with an intense therapeutic drug monitoring (TDM) to allow the attainment of therapeutic serum concentrations (37).

Vancomycin is a hydrophilic and moderately protein-bound agent and, given the narrow therapeutic window and the risk of nephrotoxicity, its PK profile has been extensively evaluated both in *in vitro* and *in vivo* settings (25–27). Indeed, targeting the ratio of the area under the vancomycin concentration–time curve over a 24-h period to the minimum inhibitory concentration of the bacteria is crucial, especially among critically ill patients (1, 38). Based on previous neonatal studies, vancomycin has shown an increased volume of distribution (Vd) and a decreased clearance (Cl) during ECMO, resulting in prolonged half-life (25–27). However, these findings were related to older roller pump-based systems, whereas data on centrifugal pump-based circuits are limited. *In vitro* studies on adult contemporary circuits have revealed either steady vancomycin levels or minimal loss over 24 and 48 h (22, 39). In contrast, we have observed a substantial decay of vancomycin, especially within the first 30 min. These results are in line with our previous *in vitro* findings on neonatal centrifugal-based circuits (18) (Table 2). The discrepancy among these results may reflect the heterogeneity of experimental settings and highlights the need for a better understanding of drug–circuit interactions, as new materials and coatings become available. A matched-cohort study conducted in adult critically ill patients on ECMO receiving continuous infusion of vancomycin showed comparable drug concentrations between ECMO and non-ECMO patients (40). Similarly, other clinical studies showed no PK differences of vancomycin between ECMO adults and critically ill patients not on ECMO (41, 42). The inconsistency between neonatal, pediatric, and adult findings may originate from either ECMO circuit-related factors or (patho-) physiological determinants, as the priming fluid impacts more on neonatal circulating blood volume compared to adults. Pending new data from pediatric clinical PK of vancomycin, it is currently recommended to increase the loading dose and to perform TDM, with target trough plasma levels between 15 and 20 μg/ml (24, 37, 43–45).

**Table 2:** Comparison of mean drug recovery at 24 h (%) between centrifugal pump-based *in vitro* studies.

	**Current experiment**					**Evidence from centrifugal-based systems**			**Physico-chemical data**		
**Drugs**	**MLP *n* = 3**	**ML *n* = 2**	**iLA *n* = 2**	**Xlung *n* = 2**	**Spontaneous drug degradation**	**Medos n Wildschut (18) *n* = 2 180 min**	**Quadrox a Shekar (22) *n* = 4 24 h**	**Quadrox a Lemaitre (39) *n* = 3 24 h**	**LogP**	**PB %**	**Vd L/kg**
VOR	17	20	19	19	0.8	-	-	-	1.8	58	4.6
VAN	60	73	60	48	2.4	67.1^*^	90	100	−3.1	50	0.4–1
Volume (ml)	250	310	400	480	-	200	670	800			

* *Recovery at 180 min. iLA, iLAActivve® MLP, MiniLung® petite; ML, MiniLung® PB, protein binding; VAN, vancomycin; Vd, volume of distribution, VOR, voriconazole, XL, Xlung®*.

Although spontaneous drug degradation was negligible for both voriconazole and vancomycin, it is methodologically rigorous to collect a dedicated sample on purpose, as the degradation may be relevant for certain drugs undergoing high spontaneous decay over time, such as midazolam (30). Moreover, according to standard practice, we have measured the total concentration of drugs, while in ECMO patients, the detection of free drug concentration could be worth exploring.

We acknowledge that our findings are not directly transferable to the bedside for a number of reasons. The number of circuits tested, which is limited by the high costs of the equipment required, calls for caution when interpreting the results to adapt the *in vitro* PK knowledge to clinical practice. Owing to the *ex vivo* setting, patient- and disease-related variables of drug disposition have not been taken into account. However, exploring the impact of circuit-related non-maturational covariates on antimicrobial PK variability is suitable to inform the ECMO compartment within the physiologically based PK modeling to develop a targeted dosing regimen during ECMO (24). Given the dearth of data related to antimicrobial drug disposition into contemporary ECMO circuits, our findings might be valuable to fill this knowledge gap in pharmacotherapy. Further efforts should be made to integrate both preclinical and clinical evidence to optimize pharmacotherapy in this vulnerable patient population by taking into account different ECMO circuit types provided by various manufacturers.

## Conclusions

These results highlight the specific impact of contemporary extracorporeal technology on antimicrobial therapy in critically ill ECMO patients. Sequestration of voriconazole in newer circuits was significant and similar to older silicone-based ones. Although to a lesser extent than voriconazole, adsorption was relevant for vancomycin. As suboptimal antibiotic therapy may lead to treatment failure, maturational toxicity, and antimicrobial resistance, TDM should be performed where available.

Meanwhile, further research is required to improve dosing accuracy and move toward tailored pharmacotherapy with the aim to deliver safe and effective ECMO management, across all age groups.

## Data Availability Statement

The raw data supporting the conclusions of this article will be made available by the authors, without undue reservation.

## Ethics Statement

The need for the institutional review board approval was waived as ethical approval was not required, according to the Dutch Law of research on human subjects. In particular, the fact that no patients were used in this *in vitro* study determined this decision.

## Author Contributions

GR was involved in the performance of experiments, acquisition, analysis, interpretation of data, writing of the first draft, and critical revision of the manuscript. GC and FM contributed to analysis, interpretation of data, and critical revision of the article. KA contributed to planning of the study, interpretation of the data, and critical revision of the article. BK performed the pharmacological analysis and critically revised the article. DT was involved in the conception and planning of the study, supervision of data collection, interpretation of the data, and critical revision of the article. EW was involved in the conception and planning of the study, performance of experiments, acquisition, analysis, interpretation of data, and critical revision of the manuscript. All authors revised the manuscript, gave final approval of the version to be submitted, and agreed to be accountable for all aspects of the work in ensuring that questions related to the accuracy or integrity of any part of the work are appropriately investigated and resolved. All authors contributed to the article and approved the submitted version.

## Conflict of Interest

The authors declare that the research was conducted in the absence of any commercial or financial relationships that could be construed as a potential conflict of interest.
